# Peroral plication of esophagus for end-stage achalasia: a systematic review

**DOI:** 10.1016/j.igie.2026.01.014

**Published:** 2026-02-02

**Authors:** Fouad Jaber, Isiah Gonzalez, Bryan Luu, Leo Kleyman, Jacob R. Moremen, Wasseem Skef

**Affiliations:** 1 Division of Gastroenterology and Hepatology, Baylor College of Medicine, Houston, Texas, USA; 2 Margaret M. and Albert B. Alkek Department of Medicine, Baylor College of Medicine, Houston, Texas, USA; 3 Division of Thoracic Surgery, University of Mississippi Medical Center, Jackson, Mississippi, USA; 4 Center for Interventional Gastroenterology at UTHealth, Houston, Texas, USA

## Abstract

**Background and Aims:**

End-stage achalasia is marked by persistent symptoms even after lower esophageal sphincter (LES)-directed therapies and may require esophagectomy, which is associated with significant adverse events. Peroral plication of the esophagus (POPE) is a novel minimally invasive, intermediate alternative. This review aimed to assess the safety and efficacy of POPE in treating end-stage achalasia.

**Methods:**

A systematic search was performed in PubMed, Embase, Web of Science, and the Cochrane Library from January 2016 to May 2025 for studies on POPE in end-stage achalasia. Primary outcomes included clinical success, defined as symptom improvement. Secondary outcomes were technical success, adverse events, need for repeat POPE, or esophagectomy. Continuous variables were summarized as means with standard deviations, whereas categorical variables were reported as pooled proportions.

**Results:**

Four studies including 45 patients with end-stage achalasia were analyzed. The cohort included 21 males (46.7%), with subtypes reported in 2 studies: 17 type I, 13 type II, and unknown or unreported subtypes (n = 15). Prior durable LES interventions included peroral endoscopic myotomy (n = 21), pneumatic dilation (n = 19), and Heller myotomy (n = 17). Regurgitation (75%) and dysphagia (65%) were the 2 most common symptoms. Pooled technical success was 100% (45/45), and pooled clinical success was 90% (37/41). Symptom improvement was observed across studies using Eckardt, gastroesophageal reflux disease Health-Related Quality of Life (GERD-HRQL), and Reflux Symptom Index scores. Eight patients required repeat POPE, and no patients underwent esophagectomy. Adverse events were observed in 7 patients (15.7%), including 2 cases of bleeding, 3 mucosal perforations, 1 readmission for pain, and 1 instance of aspiration pneumonia occurring 2 weeks after a second POPE procedure. All adverse events were managed conservatively, and no deaths were reported.

**Conclusions:**

POPE may offer a minimally invasive option for selected patients with end-stage achalasia and persistent symptoms after LES-directed therapy, but high rates of repeat procedures and considerable adverse events raise concerns about durability and safety. Larger long-term prospective studies are needed to clarify its true efficacy, safety, and ideal patient candidates.

## Introduction

Achalasia is a chronic and debilitating primary motility disorder of the esophagus characterized by degeneration of esophageal myenteric neurons leading to aperistalsis and failure of lower esophageal sphincter (LES) relaxation. Previously thought to be rare, newer studies suggest that achalasia is rather common in the United States, with 1 study suggesting incidence rates of approximately 10.5 and 26 per 100,000 person-years and prevalence rates of 18 to 162.1 per 100,000 in U.S. adults.^1^ Left untreated, long-standing esophageal outflow obstruction may cause progressive dilation, sigmoid appearance of the esophagus, or both, leading to a rare condition known as end-stage achalasia, which occurs in less than 5% of patients.^2^ Affected individuals may suffer from severe dysphagia (to both solids and liquids), regurgitation of undigested food often resulting in malnutrition, pulmonary aspiration, impairment in daily functioning, and overall quality of life.^2^

Traditional LES-directed therapeutic options are available but may be less effective in advanced achalasia. In a grossly dilated, atonic, and tortuous esophagus, alleviating the LES obstruction alone often fails to restore esophageal emptying—the esophagus remains a static reservoir with poor clearance despite treatment. For example, in 1 study of 9 patients with dilated esophagi (>7 cm), pneumatic dilation (35 mm) led to modest improvement in 5-minute timed barium esophagram (TBE)-emptying rates, from a mean (standard deviation [SD]) of 12.3 (1.8) cm to 7.8 (3.6) cm at 12-month follow-up.^3^ Similarly, Heller myotomy in this cohort demonstrated a mean rate of 79% for achieving excellent or good clinical outcomes (range 54%-100%).^4^ Peroral endoscopic myotomy (POEM) may be the preferred LES-directed therapy in this setting with 1 study of 32 patients with advanced achalasia with sigmoid-shaped esophagus demonstrating treatment success (Eckardt ≤ 3) in 30 (96.8%) patients.^5^ However, POEM might not be available because of local availability and requires significant operator experience because of technical difficulty. Indeed, patients with end-stage achalasia and megaesophagus frequently experience persistent or recurrent symptoms after standard interventions, and esophagectomy is traditionally considered the definitive salvage option in such cases.^6^ Contemporary guidelines (2018 International Society for Diseases of the Esophagus) recommend esophageal resection as a last resort for end-stage achalasia that has failed other therapies, because of the considerable morbidity of esophagectomy.^6^

Given this, a novel endoluminal therapy has been developed to address the dilated esophageal body and tortuosity called peroral plication of the esophagus (POPE). To our knowledge, Bazerbachi et al^7^ reported the first POPE case in 2017 in a patient with a sigmoid megaesophagus. The POPE technique involves endoscopic suturing to plicate the esophagus to reduce the lumen caliber and volume and straighten the esophagus. This reduction of the esophageal “sump” is intended to facilitate gravity-assisted emptying by eliminating the massive food reservoir that accumulates in end-stage disease.^8^ Early clinical experience with POPE has been encouraging but despite these theoretical advantages, there remains limited existing literature on POPE in advanced achalasia. Therefore, we aimed to systematically review the data on the clinical efficacy and safety of POPE in end-stage achalasia.

## Methodology

### Protocol registration

The aims, eligibility criteria, and analytical approach of this review were predefined and registered with the International Prospective Register of Systematic Reviews (registration no. CRD420251002971). The review was carried out in accordance with the Preferred Reporting Items for Systematic reviews and Meta-Analyses (PRISMA) guidelines.^9^

### Search strategy and study selections

Two independent reviewers (B.L. and I.G.) conducted a comprehensive search of studies reporting on POPE for achalasia or megaesophagus, covering the period from 2016 (the year before the first published POPE case report^7^) to May 2025. The search was performed across multiple databases, including PubMed, Embase, Web of Science, and the Cochrane Library. Search terms included “peroral plication,” “POPE,” “esophagus,” “achalasia,” “end-stage achalasia,” “megaesophagus,” “esophagoplasty,” and related terms (Supplemental Table 1, available online at www.igiejournal.org). Only articles published in the English language were considered. Reference lists of included studies were also reviewed to ensure a comprehensive search. Additional independent reviewers (F.J. and W.S.) reviewed and screened all selected studies, resolving disagreements and confirming final inclusion decisions.

### Eligibility criteria

This systematic review included published studies that focused on patients undergoing POPE, particularly those with achalasia. Studies were required to provide information on clinical presentation, procedural technique, outcomes, and patient demographics. Eligible studies included case series, retrospective or prospective designs, abstracts, or full-text articles reporting procedural and/or clinical outcomes of POPE. Editorials, unstructured narrative reviews, animal studies, and abstracts lacking outcome data were excluded.

### Data extraction

Data were extracted using a predesigned data extraction sheet. Information collected included study characteristics (author, year, country, and study design), patient demographics, indications for POPE, procedural details, and clinical outcomes. Outcomes of interest were safety (adverse events, mortality), technical feasibility (technical success, procedure time), and clinical efficacy, including symptoms improvement, Eckardt score, TBE, gastroesophageal reflux disease Health-Related Quality of Life (GERD-HRQL), and Reflux Symptom Index (RSI) scores.

### Data synthesis and analysis

Given the heterogeneity in study designs and outcome measures, a narrative synthesis approach was used. A quantitative data synthesis (meta-analysis) and further subgroup analyses were not performed. Included studies were descriptively summarized, and findings were organized into tables reflecting baseline characteristics, procedural details, and clinical outcomes. Because the primary objective of this review was to provide a descriptive overview, data were analyzed using descriptive statistics. Continuous variables were expressed as means with SDs, whereas categorical and binary variables were reported as pooled proportions and percentages.

### Quality assessment of the included studies

Two reviewers (F.J. and I.G.) independently evaluated study quality using the Newcastle-Ottawa Scale for cohort studies (Supplemental Table 2, available online at www.igiejournal.org). This tool assesses key methodological elements, including cohort representativeness, exposure assessment, baseline outcome status, outcome determination, and follow-up adequacy. Discrepancies between reviewers were resolved through consensus. Studies scoring above 5 were classified as high quality, scores between 3 and 5 as moderate quality, and scores below 3 as low quality. In addition, the PRISMA checklist was completed to ensure adherence to reporting standards for observational systematic reviews (Supplemental Tables 3 and 4, available online at www.igiejournal.org).

## Results

The initial search yielded 898 articles, from which 41 duplicates were removed. The remaining 857 titles and abstracts were screened for eligibility. Of these, 849 studies were excluded for not meeting the predefined inclusion criteria. Although the first reported POPE case was published in 2017,^7^ it was excluded from this review because it was later incorporated into the cohort described by Alexander et al.^10^ Similarly, the study by Hedberg et al^11^ was excluded because its cases were included in the analysis by Crafts et al.^8^ Ultimately, 4 cohort studies/case series^8^^,^^10^^,^^12^^,^^13^ published between 2016 and 2025 were included in this review, all originating from the United States. Three studies were available as full-text publications, whereas 1 was presented in abstract form. The study selection process is outlined in Figure 1.

**Figure 1:**
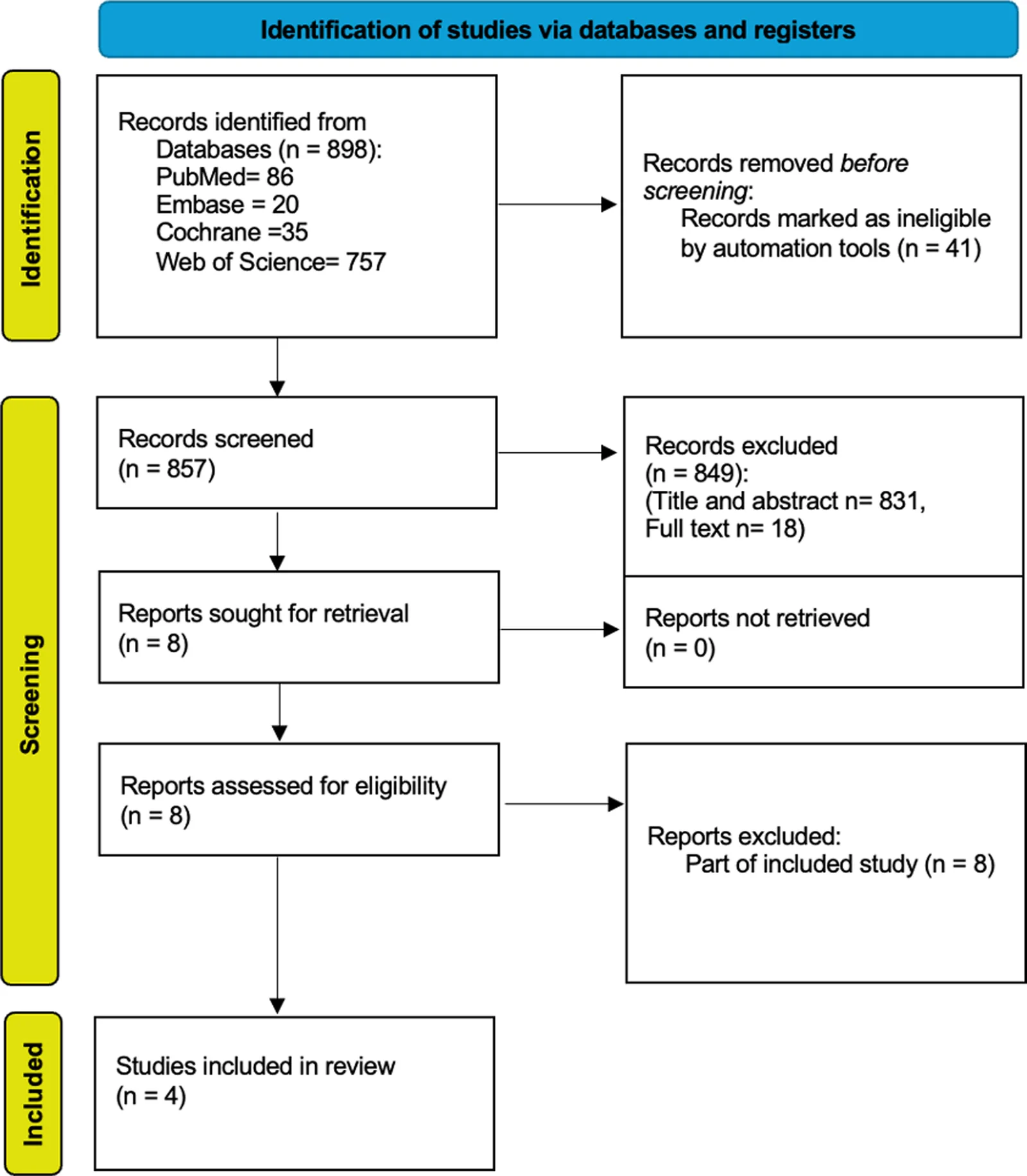
Preferred Reporting Items for Systematic reviews and Meta-Analyses flow diagram.

### Baseline characteristics of included studies and patients

All 4 included studies^8^^,^^10^^,^^12^^,^^13^ primarily focused on patients with end-stage achalasia as the indication for POPE, with the exception of Crafts et al,^8^ which also included patients with a dilated gastric conduit after esophagectomy. For the purposes of this review, however, only patients with achalasia were included in the final analysis. Andalib et al^13^ included patients with post-POEM megaesophagus, whereas Bateman et al^12^ enrolled patients with symptomatic megaesophagus with confirmed LES-directed therapy adequacy who declined esophagectomy. Alexander's et al study^10^ involved patients with end-stage achalasia with sump formation, with a preprocedural evaluation of LES to assess previous LES-directed therapy adequacy.

A total of 45 patients with end-stage achalasia were identified across the studies, of whom 21 (46.7%) were male. Achalasia subtypes were reported in 3 studies: 17 had type I, 13 had type II, and the remaining patients had unknown subtypes. The mean body mass index was reported in 2 studies: 29.1 kg/m^2^ in Bateman et al^12^ and 24 kg/m^2^ (range 18-35) in Alexander et al.^10^ Ethnicity was reported in only 1 study,^12^ which documented a 50:50 distribution between Caucasian and African American patients.

Table 1 summarizes the baseline demographics, clinical presentation, and prior interventions for the included patients. Among the 45 patients, almost all had undergone a durable LES intervention before POPE. Andalib et al^13^ included patients with achalasia who had previously undergone POEM, with 2 also having had pneumatic dilation. Bateman et al^12^ reported 1 POEM, 2 pneumatic dilations, and 3 surgical myotomies. In the study by Crafts et al,^8^ 3 patients had undergone pneumatic dilation, 4 had prior Heller myotomy, and others had received treatments such as botulinum toxin injection, recurrent stent placement, or transthoracic myotomy. In Alexander et al,^10^ all 12 patients had at least 1 prior 30-mm pneumatic dilation before POPE; in addition, 1 had undergone POEM, 2 had received botulinum toxin injections, and 10 had a history of surgical myotomy. Presenting symptoms were detailed in 2 studies, with regurgitation being the most common symptom (15/20, 75%), followed by dysphagia (13/20, 65%). Other presenting symptoms included chest pain, weight loss, aspiration pneumonia, heartburn, and food impaction.

**Table 1:** Baseline characteristics of the included studies

Variable	Andalib et al^13^	Bateman et al^12^	Crafts et al^8^	Alexander et al^10^
Study design	Retrospective, multicenter (3 centers)	Retrospective, single-center	Retrospective, single-center	Retrospective, single-center
Type of study	Abstract	Full	Full	Full
Study period	August 2022-June 2022	2023	2019-2023	January 2016-April 2024
No. of patients with achalasia	19	6	8	12
Male, n (%)	8 (42%)	2 (33.4%)	5 (62.5%)	6 (50%)
Age (years), mean (SD or range)	71	NR	60 ± 17	66 (range: 28-86)
BMI (mean)	NR	29.11	NR	24 (range: 18-35)
Indication for POPE	Post-POEM megaesophagus	Post–durable LES intervention with symptomatic megaesophagus	End-stage esophagus from achalasia	End-stage achalasia with sump formation
LES assessment before POPE	NR	Endoluminal functional imaging probe (EndoFLIP, Medtronic Inc, Minneapolis, Minn, USA)	Endoscopy and/or esophagram	TBE and EGD
Time since achalasia diagnosis, years (range)	NR	NA	NR	26 (5-47)
Mean esophagus diameter, mm, (range)	54 (45-63)	NR	NR	61 (47-83)
Sump height, mm, (range)	NR	NR	NR	18 (11-22)
Sump width, mm, (range)	NR	NR	NR	65 (20-83)
Achalasia type(s)	type I: 6, type II: 13	NR	type I: 7 unknown: 1	type I: 4 unknown: 8
Prior interventions (n)				
POEM	19	1	0	1
Botox	0	0	Yes (unspecified)	2
Pneumatic dilation	2	2	3	12
Heller myotomy	0	3	4	10
Presenting symptoms, n (%)				
Dysphagia	NR	NR	4 (50%)	9 (75%)
Aspiration pneumonia	NR	NR	2 (25%)	2 (17%)
Heartburn	NR	NR	3 (37.5%)	0 (0%)
Food impaction	NR	NR	1 (12.5%)	0 (0%)
Chest pain	NR	NR	2 (25%)	3 (25%)
Regurgitation	NR	NR	7 (87.5%)	8 (67%)
Weight loss	NR	NR	1 (12.5%)	4 (33%)

*BMI*, Body mass index; *EGD*, esophagogastroduodenoscopy; *LES*, lower esophageal sphincter; *NR*, not reported; *POEM*, peroral endoscopic myotomy; *POPE*, peroral plication of the esophagus; *SD*, standard deviation; *TBE*, timed barium esophagram.

### Preprocedure details

The mean time from achalasia diagnosis to POPE was reported in 1 study, with an average of 26 years (range 5-47). The mean preintervention esophageal diameter was noted in 2 studies: 54 mm (range 45-63) in the Andalib study^13^ and 61 mm (range 47-83) in the Alexander et al study.^10^ Sump dimensions were reported only by Alexander et al,^10^ with a mean height of 18 mm (range 11-22) and width of 65 mm (range 20-83). The exclusion of the need for further LES therapy before POPE was addressed in 3 studies. In Bateman et al,^12^ all patients underwent pre-POPE functional lumen imaging probe measurement. In Crafts et al,^8^ all patients had preoperative endoscopy, imaging, or both, with 5 receiving preoperative contrast esophagrams. In Alexander et al,^10^ both TBE and esophagogastroduodenoscopy were performed pre-POPE, and all patients had previously undergone at least one 30-mm pneumatic dilation.

### Technical aspects of POPE

Table 2 summarizes the technical aspects of the POPE procedure across the included studies. The use of an endoscopic suturing device was explicitly reported in 3 studies,^8^^,^^10^^,^^12^ which used the OverStitch Endoscopic Suturing System (Boston Scientific, Marlborough, Mass, USA) loaded onto a double-channel upper endoscope. Adjunctive thermal therapy was reported in the study by Alexander et al,^10^ where argon plasma coagulation was used to guide precise suture placement during the POPE procedure.

**Table 2:** Technical aspects of POPE across studies

Variable	Andalib et al^13^	Bateman et al^12^	Crafts et al^8^	Alexander et al^10^
Endoscopic suturing device	NR	OverStitch Endoscopic Suturing System (Boston Scientific, Marlborough, Mass, USA) loaded onto a double-channel upper endoscope	OverStitch Endoscopic Suturing System loaded onto a double-channel upper endoscope	OverStitch Endoscopic Suturing System loaded onto a double-channel upper endoscope
Adjunct thermal therapy (eg, APC)	NR	NR	NR	APC dots used as visual markers for plication
No. of sutures, mean (range)	NR	3.33 (2-7)	NR	4.5 (3-7)
No. of plications	NR	NR	Median (IQR): 3 (2-4)	NR
Suture technique	NR	Starting at the most distal area of the sump, sutures placed anterior to posterior in a distal-to-proximal direction	Distal area of sump proceeding proximally until sump eliminated; triangular pattern evolved into U-stitch pattern	Distal-to-proximal and in a running triangular suture pattern until sump collapsed
Procedure time, minutes, mean (SD or range)	46.5 (range: 40-54)	96.8 (range: 77-152)	66 (SD: 44)	57 (range: 36-79)

*APC*, Argon plasma coagulation; *IQR*, interquartile range; *NR*, not reported; *POPE*, peroral plication of the esophagus; *SD*, standard deviation.

The number of plications varied, with Bateman et al^12^ reporting a mean of 3.33 sutures (range 2-7), Crafts et al^8^ reporting a median of 3 (range 2-4) with an additional 2 to 4 sutures during repeat procedures, and Alexander et al^10^ reporting a mean of 4.5 (range 2-7) for index POPE and 5.2 (range 4-8) for repeat POPE. Details regarding the number of bites per plication were not available in any study. The suturing plan began at the sump and proceeded proximally; suture patterns were heterogeneous with Crafts et al^8^ describing a transition from a triangular to a U-stitch configuration in later cases, and Alexander et al^10^ used a consistent running triangular suture pattern alternating from the left to the right lateral wall of the sump.

Reported procedure times varied across studies, ranging from a mean of 46.5 minutes in Andalib et al^13^ (range: 40-54) to 96.8 minutes in Bateman et al^12^ (range: 77-152). Crafts et al^8^ reported a mean of 66 minutes (SD: 44), whereas Alexander et al^10^ documented a mean of 57 minutes for the index POPE (range: 36-84) and 57 minutes for repeat POPE (range: 36-78).

### Follow-up

The follow-up time varied among studies (Table 3). Andalib et al^13^ followed patients for a median of 13.5 months (range 5-15), Bateman et al^12^ for 12 months, Crafts et al^8^ had a median follow-up period of 4.6 months, and Alexander et al^10^ had a mean follow-up of 48 months (range 12-72).

**Table 3:** Clinical outcomes of the included studies

Variable	Andalib et al^13^	Bateman et al^12^	Crafts et al^8^	Alexander et al^10^
Technical success (*n*/*N*)	19/19 (100%)	6/6 (100%)	8/8 (100%)	12/12 (100%)
Clinical success (*n*/*N*)	19/19 (100%)	NA	9/10∗ (90%)	9/12 (75%)
Follow-up duration (months)	13.5 (median); range: 5-15	overall: 6-12	4.6 (median)	48 (mean); range: 12-72
Improvement in sump formation	NR	6/6	NA	9/9
Second POPE (*n*/*N*)	NR	3/6	2/8	6/12
Mean time to second POPE (months)	NR	11.6 (range: 9-17)	NR	36 (range: 12-72)
Third POPE (*n*/*N*)	NR	0/6	NR	4/12
Requiring esophagectomy	NR	0	0	0
Eckardt score, mean (SD)	9.15 → 2.2 (*P* < .0001)	6.17 → 1.83 (*P* =.046) (6-12 weeks) 6.17→ 3.5 (*P* = .072) (6-12 months)	3.8 (3.1) → 4.0 (1.4) (6 months) 3.8 (3.1)→ 8.0 (0) (12 months)	Mean ↓ 2.5 (range: 1-4) [mean: 15 months, range: 1-48]
TBE findings	NR	↑ 3.67 cm in transverse diameter (*P* < .001)	1 patient: ↓ 1.7-cm column heights; 1 no change	↓ transverse diameter 89% (mean: 16 months [0-49]) Sump recurrence 1/7 (14%) (mean: 36 months [9-80])
GERD-HRQL, mean (SD)	NR	20.17 → 10.17 (*P* = .052) (6-12 weeks) 20.17→ 7.83 (*P* = .053) (6-12 months)	13.2 (10.5) → 15.5 (12) (6 months) 13.2 (10.5) → 8.0 (0) (12 months)	NR
RSI (mean)	NR	17.83 → 7.67 (*P* = .107) (6-12 weeks) 17.83→ 9.0 (*P* = .208) (6-12 months)	19.0 (10.8) → 15.5 (6.4) (6 months) 19.0 (10.8) → 35 (0) (12 months)	NR
Length of hospital stay, median (IQR)	NR	NA (no patients required hospitalization)	0 (0-1)	0 (0-1)
Disrupted plication at repeat POPE	NR	3/6	NR	10/10

*GERD-HRQL*, Gastroesophageal reflux disease Health-Related Quality of Life; *IQR*, interquartile range; *n*, number of cases with the stated outcome; *N*, total number of cases evaluated; *NA*, not applicable; *NR*, not reported; *POPE*, peroral plication of the esophagus; *RSI*, Reflux Symptom Index; *SD*, standard deviation; *TBE*, timed barium esophagram.

∗ *n* = 10 cases of 8 patients; factors redo POPE procedure.

### Efficacy and clinical outcomes of POPE

Table 3 summarizes the clinical and efficacy outcomes across all the included studies.

#### Technical and clinical success

Across the 4 included studies, technical success was reported at 100% in all studies. Clinical success was reported in 3 studies with an average of 90% (37/41): Andalib et al^13^ (19/19, 100%), Crafts et al^8^ (9/10, 90%), and Alexander et al^10^ (9/12, 75%), whereas Bateman et al^12^ did not report this outcome (Table 4).

**Table 4:** Summary of the outcomes

Total patients (n = 45)				
Variable	Pooled studies	Mean	Range	Pooled proportion
Male	^8^^,^^10^^,^^12^^,^^13^	No./total (%): 21/45 (46.7%)		
Age (years)	^8^^,^^10^^,^^13^	68.3	28-86	
BMI	^10^^,^^14^	26.05	18-35	

Variable	Pooled studies	Total no. of events	Total no. of participants at follow-up	Pooled proportion

Technical success	^8^^,^^10^^,^^12^^,^^13^	45	45	100%
Clinical success	^8^^,^^10^^,^^13^	35	39	90%
Total adverse events	^8^^,^^10^^,^^12^^,^^13^	7	45	15.6%
Bleeding	^8^^,^^10^^,^^12^^,^^13^	2	45	4.4%
Perforation	^8^^,^^10^^,^^12^^,^^13^	3	45	6.7%
Aspiration pneumonia	^8^^,^^10^^,^^12^^,^^13^	1	45	2.2%
Pain	^8^^,^^10^^,^^12^^,^^13^	1	45	2.2%
Mortality	^8^^,^^10^^,^^12^^,^^13^	0	45	0%

*BMI*, Body mass index.

#### Repeat POPE procedure

Regarding repeat interventions, a total of 11 patients across the included studies underwent a second POPE procedure. Bateman et al^12^ reported that 3 of 6 patients (50%) required a repeat POPE procedure, whereas Crafts et al^8^ reported 2 of 8 patients undergoing a second POPE. Alexander et al^10^ noted a higher recurrence, with 6 of 12 requiring a second POPE and 4 of 12 undergoing a third POPE. The mean time to the second POPE in Bateman et al^12^ was 11.6 months, and in Alexander's study^10^ it was 3 years. Disrupted plications were seen in 3 of 6 (50%) in Bateman et al^12^ and 10 of 10 (100%) of Alexander et al's study.^10^

#### Esophagectomy

None of the patients required esophagectomy in the 3 studies^8^^,^^10^^,^^12^ that reported this outcome.

#### Radiographic assessment

Three studies^8^^,^^10^^,^^14^ reported findings of TBE after the POPE procedure. In Crafts et al,^8^ 1 patient experienced an improvement in esophageal emptying with a 1.7-cm decrease in column height, and the other had no change. In Alexander et al,^10^ 89% of patients had a reduction in transverse diameter at a mean follow-up of 16 months. Sump size was only assessed by Alexander et al,^10^ with 9 of 9 patients (100%) demonstrating radiologic improvement and 1 of 7 patients (14%) exhibiting sump recurrence at a mean follow-up of 36 months. Bateman et al^12^ reported (in their abstract) that TBE comparison showed an average increase of 3.67 cm in the maximum transverse esophageal diameter (*P* < .001; confidence interval, −1.22-0.495), despite visual improvement in sump formation and luminal narrowing. The authors attributed this discrepancy to inconsistencies in the radiologist's measurement location.

#### Validated symptom scores

##### Eckardt score

The Eckardt score was reported in all 4 studies. Andalib et al^13^ reported a significant decrease from 9.15 to 2.2 (*P* < .0001). Bateman et al^12^ reported a significant short-term improvement from a mean score of 6.17 to 1.83 (*P* = .046), which persisted on medium-term follow-up with a mean score of 3.5 at 6 to 12 months (*P* = .072). Of note, Bateman et al^12^ defined short-term outcomes as those assessed at 6 to 12 weeks and midterm outcomes at 6 to 12 months. Crafts et al^8^ did not show improvement in Eckardt with an increase from a mean of 3.8 (SD = 3.1) to 8 in 12 months, albeit with only 1 patient completing 12-month Eckardt scores. Alexander et al^10^ demonstrated improvement in the Eckardt score with a mean decrease of 2.5 (range: 1-4) over a mean of 15-month follow-up period.

##### GERD-HRQL and RSI

The GERD-HRQL and RSI were reported by Bateman et al^12^ and Crafts et al.^8^ Bateman et al^12^ reported a reduction in mean GERD-HRQL from 20.17 to 10.17 in the short term (*P* = .052) and 7.83 in the medium term (*P* = .053). Likewise, a reduction in RSI was reported from a mean of 17.83 to 7.67 on short-term follow-up (*P* = .107) and 9.0 on medium-term follow-up (*P* = .208). Of note, Bateman et al^12^ defined short-term as 6 to 12 weeks, whereas medium-term was defined as 6 to 12 months.

Crafts et al^8^ reported a reduction in GERD-HRQL from a mean of 13.2 (SD = 10.5) to 8.0 at 12 months. The RSI in Crafts et al^8^ increased from a mean of 19.0 (SD = 10.8) to 35 at 12 months.

#### Length of stay

Length of hospital stay was reported in 3 studies.^8^^,^^10^^,^^12^ No patients in Bateman et al^12^ required hospitalization. Crafts et al^8^ documented a median stay of 0 days (interquartile range: 0-1), indicating that most patients were discharged the same day or after 1 night of observation. Alexander et al^10^ reported that following the index POPE, 8 patients were discharged on the same day, and 4 required overnight observation. Among the 10 patients who underwent repeat POPE in the same study, 7 were discharged on the same day, and 3 were admitted overnight for observation.

#### Adverse events

Regarding adverse events, a total of 7 (15.7%) adverse events were reported across all 4 studies with a range varying from 8.3% to 21% (Table 5). Andalib et al^13^ reported 4 adverse events in total: 1 bleeding event treated with coagulation, and 3 perforations treated with clips. Bateman et al^12^ noted 1 adverse event: 1 bleeding event requiring repeat endoscopy without intervention or transfusion. Crafts et al^8^ reported 1 adverse event, which was a readmission for pain management. Alexander et al^10^ reported that 1 patient experienced aspiration pneumonia 2 weeks after a repeat POPE, requiring hospitalization and intravenous antibiotics. The patient was discharged home without any long-standing adverse events. No severe or fatal adverse events were reported.

**Table 5:** Safety outcomes of the included studies

Study	Andalib et al^13^	Bateman et al^12^	Crafts et al^8^	Alexander et al^10^
Total patients	19	6	8	12
Mortality	0	0	0	0
Adverse events				
Aspiration pneumonia	0	0	0	1
Bleeding	1 (treated with coagulation)	1 (repeat endoscopy without intervention)	0	0
Perforation	3 (treated with clips)	0	0	0
Readmission	NR	NR	1 (for pain, managed conservatively)	1 (pneumonia)
Total adverse events	4 (21%)	1 (16.7%)	1 (12.5%)	1 (8.3%)

*NR*, Not reported.

### Quality of the included studies

All included cohort studies were rated as high quality according to the Newcastle-Ottawa Scale (Supplemental Table 2, available online at www.igiejournal.org).

## Discussion

We conducted a systematic review of the available literature on POPE in end-stage achalasia and found that among 4 observational studies with 45 patients, the technical success rate was 100%. Moreover, symptomatic improvement was propitious with a pooled clinical success rate of 90.2% (range: 75%-100%). The safety record was acceptable, with adverse events occurring in 7 of 45 patients (15.6%)—all of which were managed endoscopically or conservatively.

For patients with end-stage achalasia not responding to LES-directed treatments, esophagectomy has long been regarded as the preferred salvage therapy.^15^ Although an appropriate option for patients with end-stage achalasia who have failed other less-invasive therapies, esophagectomy is still associated with significant morbidity and mortality. In the largest series (n = 93) of patients undergoing esophagectomy for achalasia, major adverse events included anastomotic leak (10%), recurrent laryngeal nerve injury (5%), delayed mediastinal bleeding requiring thoracotomy (2%), and chylothorax (2%). Moreover, the mortality rate was 2 of 93 patients (2%).^16^ Esophagectomy is also associated with longer-term adverse events, including nocturnal regurgitation, dumping symptoms, and anastomotic stricture.^17^ In contrast, POPE offers an organ-sparing approach with a better safety profile and promising short-term efficacy, thereby positioning it as a preferred intermediate option between durable LES therapy and esophagectomy in end-stage achalasia. However, careful patient selection is key, and confirming adequacy of myotomy with high-resolution manometry or endoluminal functional lumen imaging probe^18^ is an important prerequisite. Furthermore, scintigraphy^19^ or barium esophagraphy should be considered to evaluate the sump and confirm that it is a culprit of ongoing symptoms rather than residual esophagogastric outflow obstruction. Other anatomical considerations include the severity of esophageal dilation, degree of tortuosity, size of sump formation, and degree of mucosal stasis changes.

It is important to note that across the reported studies, a second POPE was required in 11 of 26 patients, with 1 study reporting a third POPE in 4 of 12 patients. These high reintervention rates are notable and suggest that symptom relief may be temporary for a significant subset of patients. This likely is related to the nature of end-stage achalasia, whereby stasis-related inflammation, and a dilated and tortuous lumen, creates a challenging environment to maintain durable plications. Notably, disrupted or loosened plications were documented in both Bateman et al^12^ and Alexander et al,^10^ suggesting that mechanical fatigue of sutures is common. Moreover, the POPE technique itself is not yet standardized—variation in suture pattern, number of bites, tissue-capture depth, and use of adjunctive thermal therapy may all influence durability. In addition, it is important to note that 3 perforations were documented, which is significant. This likely reflects the severity of mucosal stasis changes and friability of the esophageal tissue—notably all were successfully treated with endoscopic clips and managed without clinical sequelae.

Our study has several important strengths, which include a meticulous systematic review of the literature with diligence to avoid duplicates. Furthermore, we focused our search strategy on achalasia only to avoid heterogeneity, given that this technique can also be used to improve emptying in the gastric conduit. We also contend our results are generalizable, given that the included patients are representative of patients who undergo POPE with regard to baseline demographics and clinical variables. However, our review is limited by small sample sizes, lack of randomized studies, and inconsistency in reported outcome variables. Second, the studies included had relatively short follow-up durations, and long-term data are needed to determine the durability of these findings. Finally, given that we had only 4 studies, we were unable to perform a comprehensive meta-analysis.

In conclusion, POPE represents a potential minimally invasive option for carefully selected patients with end-stage achalasia and refractory symptoms after LES-directed therapy, especially if they are unwilling or unable to undergo esophagectomy. However, optimal procedure technique, clinical end points, and long-term durability remain unknown, and further investigations are needed to better understand how it fits within the treatment algorithm of advanced achalasia.

## Patient consent

Patient consent was not required because this was a systematic review of previously published studies and no identifiable patient information is reported.

## Disclosure

All authors disclosed no financial relationships.

## Figures and Tables

**Supplementary Table 1:** Search strategy

Database	Search terms	Search field	Search results
PubMed	(“advanced achalasia" OR “sigmoid achalasia" OR “sigmoid esophagus" OR “megaesophagus") AND (“peroral endoscopic esophagoplasty" OR “endoscopic esophagoplasty" OR “peroral plication of the esophagus")	All fields	86
Cochrane	(“advanced achalasia" OR “sigmoid achalasia" OR “sigmoid esophagus" OR “megaesophagus") AND (“peroral endoscopic esophagoplasty" OR “endoscopic esophagoplasty" OR “peroral plication of the esophagus")	All fields	35
Web of Science	(“advanced achalasia" OR “sigmoid achalasia" OR “sigmoid esophagus" OR “megaesophagus") AND (“peroral endoscopic esophagoplasty" OR “endoscopic esophagoplasty" OR “peroral plication of the esophagus")	All fields	757
Embase	**#1.**"advanced achalasia":ti,ab,kw OR “sigmoid achalasia":ti,ab,kw OR “sigmoid esophagus":ti,ab,kw OR “megaesophagus":ti,ab,kw **#2.**"peroral endoscopic esophagoplasty":ti,ab,kw OR “endoscopic esophagoplasty":ti,ab,kw OR “peroral plication of the esophagus":ti,ab,kw **#3.**#1 AND #2	All fields	20

**Supplementary Table 2:** Quality assessment of studies using Newcastle-Ottawa Scale

Study/year	Selection				Comparability	Outcomes			Score	Quality
	Representativeness of the targeted population	Cohort size	Information on outcomes	Outcome not present at start	Yes: 1, no: 0	Assessment of outcomes	Follow-up Time	Adequacy of follow-up		
	Yes: 1, no: 0	>10 patients, 1; 5-10, .5; <5, 0	Information with clarity, 1; information derived, .5	Not present,1; present, 0		Yes, 1; no, 0	Yes, 1; not mentioned, 0	All patients followed up, 1; >50% followed up, .5; <50% followed up or not mentioned, 0	Max = 8, high >5, medium 3-5, low <3	High >5, medium 3-5, low <3
Andalib 2024,^13^	1	1	1	1	0	1	1	1	7	High
Bateman 2025,^12^	1	0.5	0.5	1	0	1	1	1	6	High
Crafts 2024,^8^	1	0.5	1	1	0	1	1	1	6.5	High
Alexander 2024,^10^	1	1	1	1	0	1	1	1	7	Moderate

Values of 1, 0.5, and 0 indicate full, partial, and no credit, respectively, for each quality-assessment criterion.

**Supplementary Table 3:** PRISMA checklist for the manuscript

Section and topic	Item no.	Checklist item	Location where item is reported (page numbers)
TITLE			
Title	1	Identify the report as a systematic review.	1
ABSTRACT			
Abstract	2	See the PRISMA 2020 for Abstracts checklist.	2
INTRODUCTION			
Rationale	3	Describe the rationale for the review in the context of existing knowledge.	4
Objectives	4	Provide an explicit statement of the objective(s) or question(s) the review addresses.	4
METHODS			
Eligibility criteria	5	Specify the inclusion and exclusion criteria for the review and how studies were grouped for the syntheses.	5
Information sources	6	Specify all databases, registers, websites, organizations, reference lists, and other sources searched or consulted to identify studies. Specify the date when each source was last searched or consulted.	4
Search strategy	7	Present the full-search strategies for all databases, registers, and websites, including any filters and limits used.	4
Selection process	8	Specify the methods used to decide whether a study met the inclusion criteria of the review, including how many reviewers screened each record and each report retrieved, whether they worked independently, and if applicable, details of automation tools used in the process.	4-5
Data collection process	9	Specify the methods used to collect data from reports, including how many reviewers collected data from each report, whether they worked independently, any processes for obtaining or confirming data from study investigators, and if applicable, details of automation tools used in the process.	5
Data items	10a	List and define all outcomes for which data were sought. Specify whether all results that were compatible with each outcome domain in each study were sought (eg, for all measures, time points, analyses), and if not, the methods used to decide which results to collect.	5
	10b	List and define all other variables for which data were sought (eg, participant and intervention characteristics, funding sources). Describe any assumptions made about any missing or unclear information.	5
Study risk of bias assessment	11	Specify the methods used to assess risk of bias in the included studies, including details of the tool(s) used, how many reviewers assessed each study, and whether they worked independently, and if applicable, details of automation tools used in the process.	5
Effect measures	12	Specify for each outcome the effect measure(s) (eg, risk ratio, mean difference) used in the synthesis or presentation of results.	5
Synthesis methods	13a	Describe the processes used to decide which studies were eligible for each synthesis (eg, tabulating the study intervention characteristics and comparing against the planned groups for each synthesis (item no. 5).	5
	13b	Describe any methods required to prepare the data for presentation or synthesis, such as handling of missing summary statistics, or data conversions.	5
	13c	Describe any methods used to tabulate or visually display results of individual studies and syntheses.	5
	13d	Describe any methods used to synthesize results and provide a rationale for the choice(s). If meta-analysis was performed, describe the model(s), method(s) to identify the presence and extent of statistical heterogeneity, and software package(s) used.	5
	13e	Describe any methods used to explore possible causes of heterogeneity among study results (eg, subgroup analysis, meta-regression).	NA
	13f	Describe any sensitivity analyses conducted to assess robustness of the synthesized results.	NA
Reporting bias assessment	14	Describe any methods used to assess risk of bias due to missing results in a synthesis (arising from reporting biases).	8
Certainty assessment	15	Describe any methods used to assess certainty (or confidence) in the body of evidence for an outcome.	NA
RESULTS			
Study selection	16a	Describe the results of the search and selection process, from the number of records identified in the search to the number of studies included in the review, ideally using a flow diagram.	5
	16b	Cite studies that might appear to meet the inclusion criteria, but which were excluded, and explain why they were excluded.	5
Study characteristics	17	Cite each included study and present its characteristics.	5
Risk of bias in studies	18	Present assessments of risk of bias for each included study.	Supplementary Table 2
Results of individual studies	19	For all outcomes, present, for each study (a) summary statistics for each group (where appropriate) and (b) an effect estimate and its precision (eg, confidence/credible interval), ideally using structured tables or plots.	5-7
Results of syntheses	20a	For each synthesis, briefly summarise the characteristics and risk of bias among contributing studies.	Supplementary Table 2
	20b	Present results of all statistical syntheses conducted. If meta-analysis was done, present for each the summary estimate and its precision (eg, confidence/credible interval) and measures of statistical heterogeneity. If comparing groups, describe the direction of the effect.	5-7
	20c	Present results of all investigations of possible causes of heterogeneity among study results.	NA
	20d	Present results of all sensitivity analyses conducted to assess the robustness of the synthesized results.	NA
Reporting biases	21	Present assessments of risk of bias due to missing results (arising from reporting biases) for each synthesis assessed.	NA
Certainty of evidence	22	Present assessments of certainty (or confidence) in the body of evidence for each outcome assessed.	NA
DISCUSSION			
Discussion	23a	Provide a general interpretation of the results in the context of other evidence.	8
	23b	Discuss any limitations of the evidence included in the review.	8
	23c	Discuss any limitations of the review processes used.	8
	23d	Discuss implications of the results for practice, policy, and future research.	8
OTHER INFORMATION			
Registration and protocol	24a	Provide registration information for the review, including register name and registration number, or state that the review was not registered.	4
	24b	Indicate where the review protocol can be accessed, or state that a protocol was not prepared.	4
	24c	Describe and explain any amendments to information provided at registration or in the protocol.	4
Support	25	Describe sources of financial or nonfinancial support for the review, and the role of the funders or sponsors in the review.	1
Competing interests	26	Declare any competing interests of review authors.	1
Availability of data, code and other materials	27	Report which of the following are publicly available and where they can be found: template data collection forms; data extracted from included studies; data used for all analyses; analytic code; any other materials used in the review.	NA

*From:* Page MJ, McKenzie JE, Bossuyt PM, et al. The PRISMA 2020 statement: an updated guideline for reporting systematic reviews. BMJ 2021;372:n71. This work is licensed under CC BY 4.0. To view a copy of this license, visit https://creativecommons.org/licenses/by/4.0/

*NA*, Not applicable; *PRISMA*, Preferred Reporting Items for Systematic reviews and Meta-Analyses.

**Supplementary Table 4:** Preferred Reporting Items for Systematic reviews and Meta-Analyses checklist for the abstract

Section and topic	Item no.	Checklist item	Reported (yes/no)
TITLE			
Title	1	Identify the report as a systematic review.	YES
BACKGROUND			
Objectives	2	Provide an explicit statement of the main objective(s) or question(s) the review addresses.	YES
METHODS			
Eligibility criteria	3	Specify the inclusion and exclusion criteria for the review.	YES
Information sources	4	Specify the information sources (eg, databases, registers) used to identify studies and the date when each was last searched.	YES
Risk of bias	5	Specify the methods used to assess risk of bias in the included studies.	YES
Synthesis of results	6	Specify the methods used to present and synthesise results.	YES
RESULTS			
Included studies	7	Give the total number of included studies and participants and summarise relevant characteristics of studies.	YES
Synthesis of results	8	Present results for main outcomes, preferably indicating the number of included studies and participants for each. If meta-analysis was done, report the summary estimate and confidence/credible interval. If comparing groups, indicate the direction of the effect (ie, which group is favoured).	YES
DISCUSSION			
Limitations of evidence	9	Provide a brief summary of the limitations of the evidence included in the review (eg, study risk of bias, inconsistency, and imprecision).	YES
Interpretation	10	Provide a general interpretation of the results and important implications.	YES
OTHER			
Funding	11	Specify the primary source of funding for the review.	YES
Registration	12	Provide the register name and registration number.	YES
